# Retraction Note: Peripheral blood mono-nuclear cells implantation in patients with peripheral arterial disease: a pilot study for clinical and biochemical outcome of neoangiogenesis

**DOI:** 10.1186/s12893-016-0138-1

**Published:** 2016-04-28

**Authors:** Bruno Amato, Rita Compagna, Gianni Antonio Della Corte, Giovanni Martino, Tommaso Bianco, Guido Coretti, Roberto Rossi, Antonio Braucci, Giovanni Aprea, Pio Zeppa, Alessandro Puzziello, Claudio Terranova

**Affiliations:** Department of General, Geriatric, Oncologic Surgery and Advanced Technologies University of Naples Federico II, Via S. Pansini, Napoli, 5 – 801311 Italy; Department of Medicine and Surgery, University of Salerno, Salerno, Italy; Department of Medical and Surgical Sciences, University Magna Graecia, Catanzaro, Italy; Department of Molecular Medicine, Padova University Hospital, Italy, Via Giustiniani n.2, Padova, 35126 Italy

## Retraction Notice

This article [1] has been retracted by the authors due to significant overlap with a previous publications, including Tateno et al. [2] and Moriya et al. [3]. The authors apologise for failing to cite these articles. Claudio Terranova was not involved in the study and was introduced into the author list in error. All authors, including Claudio Terranova, have subsequently agreed that he did not qualify for authorship.

## Notes

The online version of the original article can be found at 10.1186/1471-2482-12-S1-S1.
